# Forecasting Compressive Strength of RHA Based Concrete Using Multi-Expression Programming

**DOI:** 10.3390/ma15113808

**Published:** 2022-05-26

**Authors:** Muhammad Nasir Amin, Kaffayatullah Khan, Muhammad Faisal Javed, Dina Yehia Zakaria Ewais, Muhammad Ghulam Qadir, Muhammad Iftikhar Faraz, Mir Waqas Alam, Anas Abdulalim Alabdullah, Muhammad Imran

**Affiliations:** 1 Department of Civil and Environmental Engineering, College of Engineering, King Faisal University, P.O. Box 380, Al-Hofuf, Al-Ahsa 31982, Saudi Arabia; kkhan@kfu.edu.sa (K.K.); 218038024@student.kfu.edu.sa (A.A.A.); 2 Department of Civil Engineering, Abbottabad Campus, COMSATS University Islamabad, Abbottabad 22060, Pakistan; arbabfaisal@cuiatd.edu.pk; 3 Structural Engineering, Faculty of Engineering and Technology, Future University in Egypt, New Cairo 11835, Egypt; dina.yehya@fue.edu.eg; 4 Department of Environmental Sciences, Abbottabad Campus, COMSATS University Islamabad, Abbottabad 22060, Pakistan; hashir785@gmail.com; 5 Department of Mechanical Engineering, College of Engineering, King Faisal University, P.O. Box 380, Al-Hofuf, Al-Ahsa 31982, Saudi Arabia; mfaraz@kfu.edu.sa; 6 Department of Physics, College of Science, King Faisal University, P.O. Box 380, Al-Hofuf, Al-Ahsa 31982, Saudi Arabia; wmir@kfu.edu.sa; 7 School of Civil and Environmental Engineering (SCEE), National University of Sciences & Technology (NUST), Islamabad 44000, Pakistan; imran.nice@nust.edu.pk

**Keywords:** rice husk ash, machine learning, waste material, external validation, compressive strength

## Abstract

Rice husk ash (RHA) is a significant pollutant produced by agricultural sectors that cause a malignant outcome to the environment. To encourage the re-use of RHA, this work used multi expression programming (MEP) to construct an empirical model for forecasting the compressive nature of concrete made with RHA (CRHA) as a cement substitute. Thus, the compressive strength of CRHA was developed comprising of 192 findings from the broad and trustworthy database obtained from literature review. The most significant characteristics, namely the specimen’s age, the percentage of RHA, the amount of cement, superplasticizer, aggregates, and the amount of water, were used as input for the modeling of CRHA. External validation, sensitivity analysis, statistical checks, and Shapley Additive Explanations (SHAP) analysis were used to evaluate the models’ performance. It was discovered that the most significant factors impacting the compressive strength of CRHA are the age of the concrete sample (AS), the amount of cement (C) and the amount of aggregate (A). The findings of this study have the potential to increase the re-use of RHA in the production of green concrete, hence promoting environmental protection and financial gain.

## 1. Introduction

**Figure 1:**
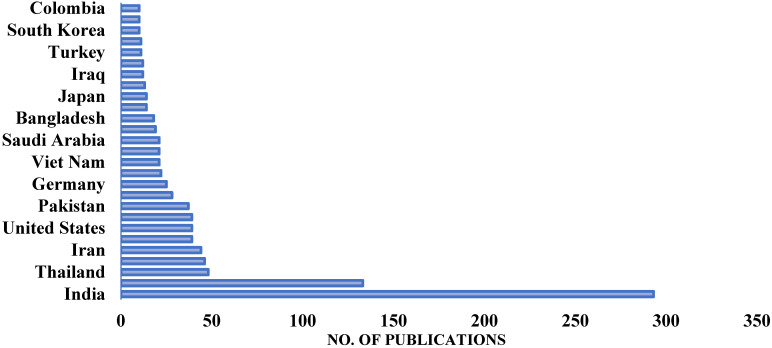
Number of publications on RHA.

**Figure 2:**
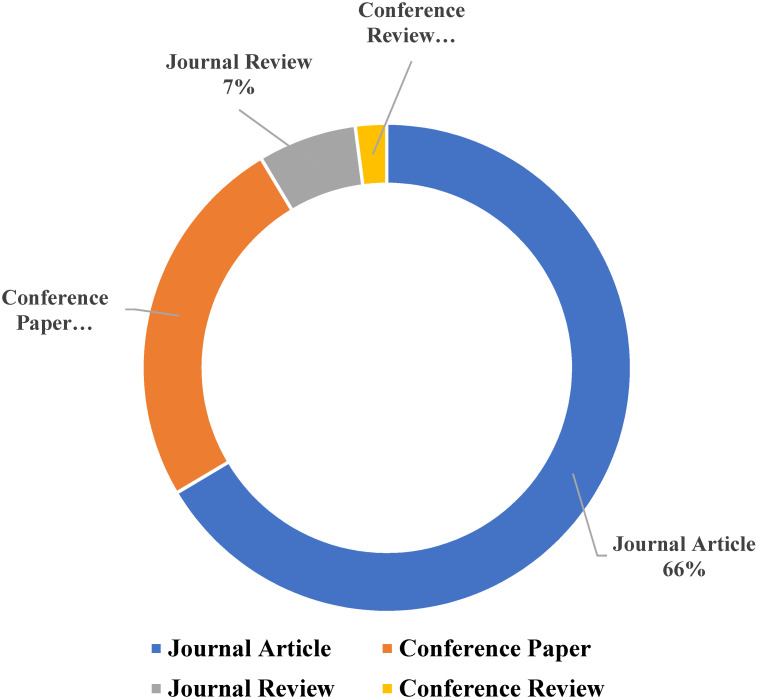
Publications of RHA in high-impact factor journals.

**Figure 3:**
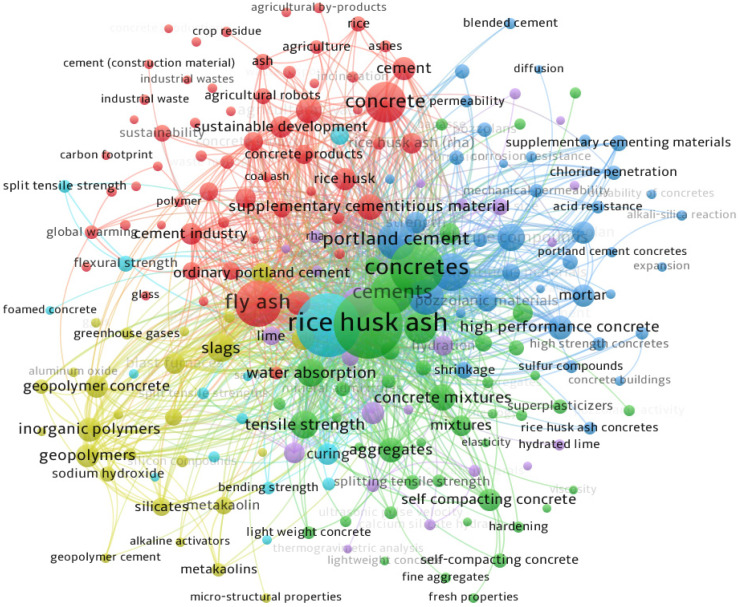
Importance of RHA in the construction industry.

Different researchers have suggested different methods to lessen the malignant impacts of the construction industry on the atmosphere. Some researchers suggested replacing the natural coarse aggregate in concrete with recycled concrete aggregate, oil palm shell aggregate, lightweight aggregate, rubber, and so on, while others suggested replacing natural sand with sugarcane bagasse ash, rice husk ash (RHA), eggshell ash, and other different types of industrial and agricultural wastes [1,2,3,4]. However, it is observed to be more beneficial if cement is replaced with concrete, as cement is the main culprit in concrete which affects the environment. The partial replacement of cement with natural pozzolanic materials, industrial wastes, and agricultural wastes has been a point of interest for different researchers for the last couple of decades [5,6]. One of the common agricultural wastes is RHA, which is highly pozzolanic and contains a high amount of silica content. RHA is a byproduct of the cultivation of rice. RHA is formed as a result of heating husks in processing industries in order to process rice paddy. Rice is one of the world’s most important food crops and is consumed in vast amounts by the global population. As of 2020/2021, it is estimated that 497.7 million tons of rice are produced globally. Therefore, RHA is prevalent in agricultural nations that produce millions of metric tons of rice annually. As it includes roughly 85–90% amorphous silica, RHA may be effectively recycled as a pozzolanic material as opposed to being discarded publicly. The use of RHA in concrete has been researched by different scientists [7,8,9]. The research on RHA is mostly conducted in Agricultural countries as shown in Figure 1. The gathered data is up to April 2022 as illustrated in Figure 1. The number of publications from India is more than twice that of any other country on RHA. Most of the research performed on RHA is published in high-impact Journals as shown in Figure 2. RHA is mainly utilized as a partial replacement of cement (as Supplementary Cementitious Material) and provides better properties than normal concrete (concrete without RHA). RHA can be used for many other purposes as shown in Figure 3, but they are out of the scope of this study. Concrete made with RHA (CRHA) is reported to be more durable and posseses higher mechanical properties when compared with normal concrete [9,10,11]. In addition, the use of RHA in concrete provides sustainability to the construction industry in two ways. First, it reduces the amount of cement (C) used, and second, it helps in the disposal of waste RHA. Furthermore, concrete made with RHA is more economical as some percentage of cement (the most expensive material in concrete) is being replaced with waste material. The behavior of RHA concrete is anomalous due to numerous factors, i.e., concrete mix design, amount of RHA used, and physical properties of concrete ingredients [12,13,14,15]. Therefore, the use of RHA requires prior experimental testing to be used in mega projects. However, the presence of reliable, trustworthy models and formulas to relate the compressive strength of RHA concrete with its ingredients may provide ease to construction engineers to use RHA concrete in their projects. The wide use of RHA concrete may help in reducing the carbon footprint of the construction industry. The use of modern computing techniques like artificial intelligence algorithms (AIA) can be used to achieve this objective.

The use of AIA is rising in every field [16,17,18,19,20,21,22,23]. AIA has distinctive features like pattern recognition and object recognition, which can be used to solve various engineering problems [24,25,26,27,28,29,30]. However, AIA is generally termed as black-box algorithms (BBA), because it does not give an insight into the problem being solved [31,32]. AIA ignores any knowledge or physical occurrences related to the subject at hand. The majority of ANN approaches lag in the development of an advanced mathematical formulation for estimating output based on input factors [33,34,35]. A correlation between input and output is referred to as an ANN-based model, and the relationship seems to be either nonlinear or based on a pre-defined structure [36,37,38]. To address these challenges, numerous evolutionary algorithms (EA) are being used to simulate concrete features, including genetic programming, convolutional neural networks (NN), and the model tree algorithm [19,39,40,41]. The advantage of EA is that they enable the production of realistic algebraic expressions, as well as a high degree of generality and prediction capabilities [1,3,4,5,6]. A few recent research have attempted to simulate the characteristics of waste foundry sand concrete using AIA. Different EA was used to create decision tree structures for the purpose of estimating the mechanical characteristics of waste foundry sand concrete [42]. Numerous influencing factors, a robust correlation coefficient, and minor arithmetical errors were obtained for the constructed models. Nevertheless, parametric research was not possible due to the linear character of decision trees, which reduces their effectiveness when applied to unknown data. Similarly, in a recent study, a genetic programming approach was used to estimate the compressive strength of waste foundry sand concrete [43]. To assess the suggested models’ dependability, parametric, and error, sensitivity analyses were conducted. However, the gene expression programming (GEP) approach has drawbacks in that it was powerless to contain a few differing datasets into the model construction process, hence limiting its application range [44]. To improve the performance of the models, the differing datapoints required to be eliminated from the set processes. Additionally, genetic algorithms (GA) program uses a solitary chromosome, and are useful when the relationship between the targeted and predicted is reasonably basic.

To overcome the drawbacks of AIA, an enhanced modeling approach known as multi-expression programming (MEP) was utilized to predict the mechanical characteristics, i.e., compressive strength of CRHA based on the most influential factors. MEP is unique in that it can encode many expressions in one computer program [45,46]. To guarantee that the models are effectively trained, a big database was compiled from the literature and subdivided into three sets: training, validation, and testing. The effectiveness of the models is assessed by using statistical error analysis, external validation, and various statistical analyses to ensure that the models are generalizable and reliable. The article is arranged as follows: a description of the MEP algorithm, a database of experimental findings, a modeling approach, results and discussion, external validation, sensitivity analysis, and lastly, a brief discussion of the conclusion and significant discoveries.

## 2. Multi-Expression Programming (MEP)

**Figure 4:**
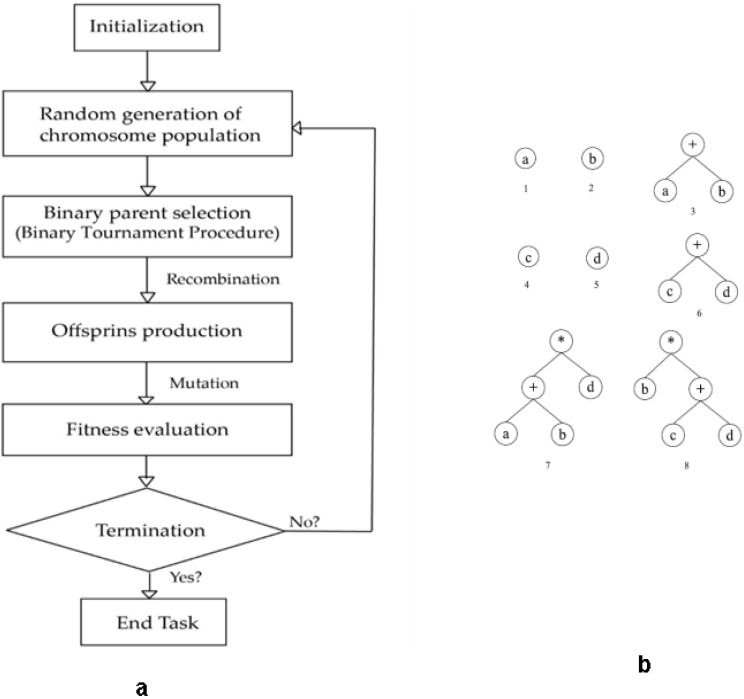
(**a**) Procedures involved in implementing MEP, (**b**) Flowchart for expressions encoded by an MEP chromosome.

The goal of a machine learning model is to produce a mathematical expression for output prediction that is accurate and practicable based on a collection of independent parameters. Koza (1992) suggested the GEP as an evolution of the GA based on Darwin’s selection concept [47]. It is important to note that the main difference between the two techniques is that in GEP, fixed-length binary strings are replaced with non-linear parse trees. Several other types of EAs have been proposed in recent years, with linearity being a key one. Individuals (chromosomes) can be modeled as variable-length entities in the case of MEP [48]. MEP simulation output may be characterized as a linear string of instructions consisting of variables or mathematical operations (functions). Figure 4 illustrates the procedures involved in implementing MEP [48]. The process of MEP starts with the initialization of functions and expressions. After that, the chromosomes population is increased randomly based on the binary selection of the connection functions as shown in Figure 4b. When the chromosomes population reach a certain point, off-springs are produced and evaluated with the help of the evaluation function. The process is terminated when the required fitness value is achieved. The MEP method evolves by creating a random chromosomal population, selecting two parents via a binary tournament, and recombination with a set cross-over frequency, the generation of two offspring through recombination of the selected parents, mutation of the offspring, and replacement of the population’s worst individuals with the best are some of the steps followed in MEP. The process is cyclical and repeats itself until convergence is attained.

Most of the research over the last decade has been on the application of artificial neural network (ANN) and GEP approaches to model the characteristics of green concrete. However, MEP has several benefits over comparable algorithms. Typically, a large database is used to represent concrete characteristics. In GEP, just a cross-over genetic operator is used, resulting in the generation of a large population of parse trees, increasing simulation time and requiring a considerable amount of memory [47,49,50]. Additionally, because GEP’s non-linear structure functions like gene expression patterns, the algorithm has a hard time proposing a simple mathematical representation for the required attribute. The integration of linear variants enables MEP to discriminate between an individual’s genotype and phenotype. Moreover, up to a certain point, the amount of genes on chromosomes improves the likelihood of GEP success. The model’s usefulness in the construction industry is limited by overfitting, which manifests itself in the predicted strength qualities in the construction industry. In fact, MEP is particularly useful when the objective expression is uncertain, as in material engineering problems where a small change in a concrete mix parameter might have a huge impact on the strength [48]. Due to the linearity of chromosomes and the encoding of numerous solutions in one chromosome in MEP, the software may search for a larger space for the output prediction. Due to the evident benefits of MEP over other EAs, accurate models in the field of civil engineering may be developed. It has been used in several research to forecast different soil properties using physical properties of soil as input parameter [34], to predict the elasticity of concrete by using mix design ratios, and to create predicting modeling for concrete columns confined with thermoplastic fiber reinforced polymer [51]. The present work used the MEP approach to develop models to predict the parameters of CRHA. Further validation of the model is made by applying various statistical checks to the model. The availability of trustworthy models will encourage the use of CRHA in the building sector since it circumvents the time-consuming testing process necessary for such an unconventional construction material. This would help to waste reduction, sustainable building, and natural resource conservation. Additionally, the provided modeling technique will pave the way for correctly modeling comparable complicated engineering processes.

## 3. Data Collection

To build a computational equation that properly predicted the compressive strength of CRHA, a database of 192 data points from the published research was employed (Table S1) [52,53,54,55,56,57,58]. The CRHA is composed of the same components: OPC, RHA, aggregates (A), water (W), and superplasticizer (SP). All mixtures obtained from the literature utilized the same type of cement with identical age of concrete (AS). The correlation matrix for the inputs and compressive strength (CS) of CRHA is shown in Table 1.

**Figure 5:**
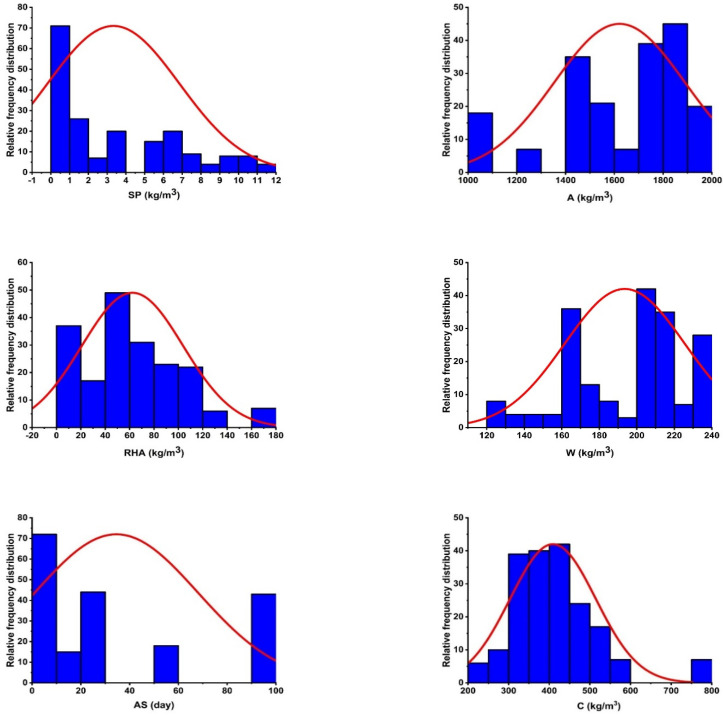

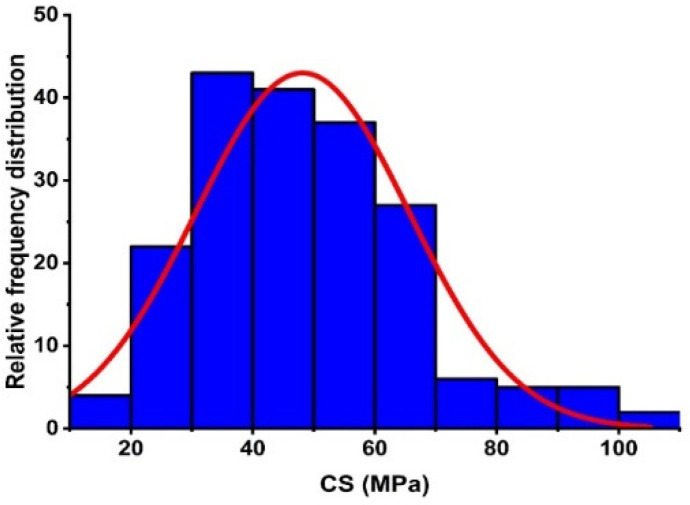
Histogram of variables used in making model.

The compressive strength of cubic specimens was converted to the compressive strength of cylinders using a conversion ratio of 0.8 [59]. The purpose of this research was to determine the compressive strength of various CRHA mixtures using MEP. As input parameters, variables such as the amount of cement (C), the amount of water (W), amount of RHA, age of concrete (AS), amount of aggregate (A), and dosage of SP were collected from the literature. Figure 5 depicts histograms for all variables utilized in this investigation. Additionally, Table 2 has a statistical description of the gathered data. The mean and median for all AS values were obtained to be 34.57 and 28, respectively. While the value of skewness is positive for all the variables except for water and fine aggregate.

## 4. Model Development

One of the objectives of this study is to develop a new formulation for the compressive strength of CRHA using the MEP model. The essential parameters recommended in the literature were used as input variables. Therefore, formulation of the compressive strength (CS) of CRHA was assumed using Equation (1) as follows:

(1) CS=f(AS,OPC,A, SP, W, RHA)

In order to develop a strong and generic model, a large number of MEP fitting parameters must be defined before modeling begins. The relevant variables are chosen in accordance with prior suggestions and a trial-and-error method. The number of programs that will develop in a population is determined by the size of the population. It would be more complex and precise to run a model with a huge population size, and it may take a long time for the model to converge. The method was begun by assuming a total of ten subpopulations. Table 3 summarizes the parameters used in the study. All these values are calculated after running several trials on different combinations as shown in Table 4. It should be noted that several parameters (like code length, connecting functions) can further increase the accuracy of the developed model, but they increase the computation time as well as the complexity of the model. Hence, they were kept at an optimum level. For simplicity in the final formulations, the function set includes the fundamental mathematical operations of multiplication, square root, natural log, subtraction, division, and addition. The number of generations indicates the amount of accuracy that the algorithm should reach before being terminated. Similarly, the rate of mutation and cross-over indicates the likelihood that the progeny will experience similar genetic processes. The incidence of cross-over varies between 50% and 95%. Numerous combinations of these parameters were tested on the data, and the optimal combination was chosen as shown in Table 4. The final parameters selected are shown in Table 3. One of the challenges with AI-based modeling is data overfitting. A model works admirably on the original data, but drastically degrades on the unseen data. To circumvent this issue, it has been proposed to test the trained model on an unknown or testing dataset. As a result, the whole database was randomly partitioned into training, validation, and testing sets. While modeling, the training and validation data were processed. The validated model is next evaluated on a third dataset, i.e., one that was not utilized to construct the model. It was assured that the distribution was uniform across all datasets. The resulting models outperformed the baseline models on all three datasets. MPX v1.0, a commercially available computer tool, was used to implement the MEP algorithm [44,45,46].

### 4.1. Shapley Additive Explanations (SHAP)

Even though numerous ML research on concrete structures have attained great accuracy in their predictions, the applicability of the ML models receives little consideration. Numerous research assesses the feature relevance for tree-based models single decision process, heuristic techniques, or model-agnostic methods [47,48]. However, these approaches are frequently impractical and skewed for EML models, particularly those with a significant bias. In this study, SHAP is utilized to demonstrate the interpretation of every input parameter. SHAP is expressed as the mean marginal contribution to a feature value over all conceivable coalitions, in accordance with the collaborative game theory. In particular, the SHAP value of a data is the mean prediction rate of samples having the characteristic minus the mean prediction value of samples lacking the feature. To enhance the interpretability of a machine learning (ML) model, its output is stated as the linear sum of its input data multiplied by their respective SHAP values.

To check the performance criteria, Root mean square error (RMSE), coefficient of correlation (R), mean absolute error (MAE), coefficient of regression (R^2^), relative root mean square error (RRMSE), relative squared error (RE), and performance index ρ (Equations (2)–(8), respectively) have been used in this study.

(2) $$\mathrm{RMSE}=\sqrt{\frac{\sum_{i=1}^{n}{\left(x_{i}-y_{i}\right)}^{2}}{n}}$$

(3) $$R=\frac{\sum_{i=1}^{n}\left(x_{i}-{\bar{x}}_{i}\right)\left(y_{i}-{\bar{y}}_{i}\right)}{\sqrt{\sum_{i=1}^{n}{\left(x_{i}-{\bar{x}}_{i}\right)}^{2}\sum_{i=1}^{n}{\left(y_{i}-{\bar{y}}_{i}\right)}^{2}}}$$

(4) $$\mathrm{MAE}=\frac{\sum_{i=1}^{n}\left|x_{i}-y_{i}\right|}{n}$$

(5) $$R^{2}=1-\frac{\sum_{i=1}^{n}{\left(x_{j}-y_{j}\right)}^{2}}{\sum_{i=1}^{n}\left(x_{j}-\bar{y}\right)}$$

(6) $$\mathrm{RRMSE}=\frac{1}{\left|\bar{e}\right|}\sqrt{\frac{\sum_{i=1}^{n}{\left(x_{i}-y_{i}\right)}^{2}}{n}}$$

(7) $$\mathrm{RE}=\frac{\sum_{i=1}^{n}{\left(x_{i}-y_{i}\right)}^{2}}{\sum_{i=1}^{n}{\left(\bar{x}-x_{i}\right)}^{2}}$$

(8) $$\rho =\frac{\mathrm{RRMSE}}{1+R}$$

(9) $$\mathrm{OBJ}=\left(\frac{n_{L}-n_{T}\;}{n}\right)\rho_{L}+2\left(\frac{n_{T}}{n}\right)\rho_{T}$$

where, $x_{i}$ and $y_{i}$ are the ith experimental and predicted output values, respectively; and denote the experimental and expected output values, respectively; and n denotes the complete number of observations. Lower values of RMSE, MAE, and higher values of R, and R^2^, as well as the pre-selected significance value, i.e., alpha (usually 0.05) from F and *t*-tests, indicate that the predictive model performs well and has a better accuracy. Additionally, it implies that the experimental and anticipated values are highly connected. Additionally, it is worth noting that a R value larger than 0.8, an R^2^ value nearer to 1, an RMSE value nearer to or equal to zero, and ρ value (0 to infinity) approaching zero all contribute to improved model calibration. Unlike the RMSE, MAE is a positive evolution metric when the original data is relatively smooth [60]. On the other hand, the normalized mean square error (NSE) runs between 0 and 1.0 (1 inclusive), with 1 regarded as the best number. Additionally, a significant issue linked with AI systems is overfitting, which occurs because of extensive training and results in higher mistakes in the testing set. As demonstrated in Equation (9), the objective function (OBF) is assessed and decreased prior to selecting the best predictive mode [61]. The OBF is used to evaluate the trained model’s performance by including changes in the error function (RRMSE) and correlation coefficient (R). A low OBF value aids in overcoming the issue of overfitting.

### 4.2. Cross-Validation Using 10 K-Fold Method

**Figure 6:**
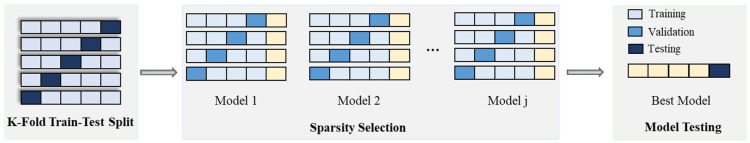
K-fold cross-validation algorithm [61].

Generally, cross-validation procedure is applied using 10 k-fold to decrease the random sampling-related distortion of training and residual set of inputs. According to the findings of Kohavi, the ten-fold validation test yields a dependable variance and the ideal computing time (Kohavi, 1995). This study employed a stratified 10 k-fold cross-validation method to evaluate the performance of a model that categorizes a given number of data samples into 10 subgroups. In each of 10 rounds of model development and validation, a separate data subset is used for testing while the remaining data subsets are used to train the model. As seen in Figure 6, the test subset is used to validate model precision. The algorithm’s precision is then reported as the average precision gained by the 10 models during ten rounds of validation.

## 5. Results and Discussion

### 5.1. MEP Analysis of CRHA

**Figure 7:**
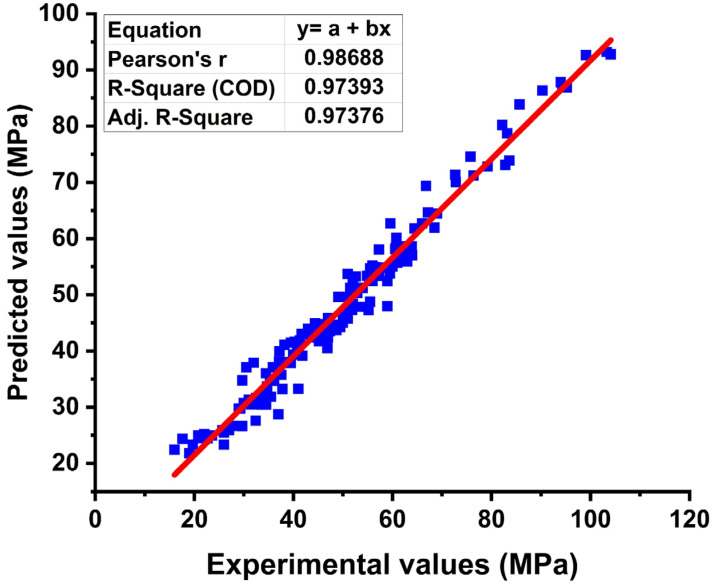
Regression analysis of training set of MEP.

**Figure 8:**
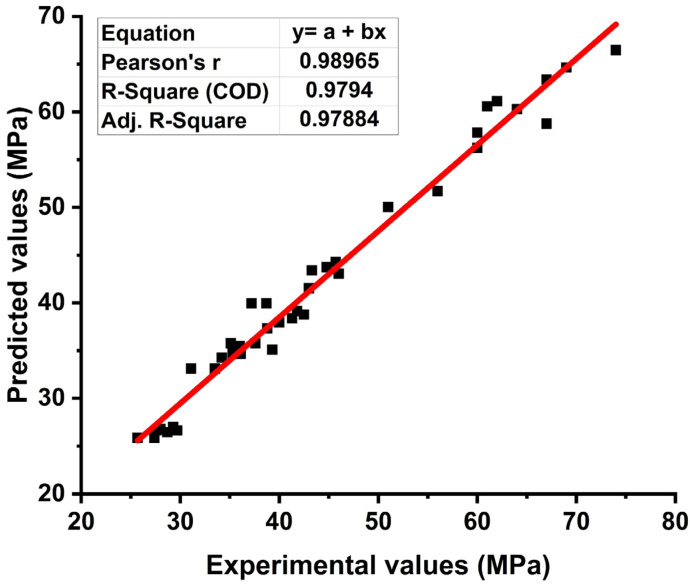
Regression analysis of testing set of MEP.

Appendix A contains the optimized MEP code for compressive strength prediction of CRHA utilizing specified input variables. The compressive strength of CRHA for the training dataset is displayed in Figure 7 along with the slope. The optimal location of the regression line is 45°, with a slope equal to 1, but it must be closer to 1 for good association. As shown in Figure 7, the proposed model accurately predicts the compressive strength of CRHA (R for the entire dataset is 0.97). Additionally, the RMSE, MAE, and the NSE for estimating the training dataset of compressive strength of CRHA are 3.98, 0.6, and 0.77, respectively. The near proximity of the points to the ideal fit and the inclusion of most points within the acceptable confidence interval demonstrates the suggested MEP model’s validity. As previously stated, R values greater than 0.8 [45] and NSE values near unity indicate that the suggested models for the compaction parameters function effectively. Figure 8 shows the compressive strength of CRHA for validation and testing set. For simplicity, both sets are combined in the Figure 8.

**Figure 9:**
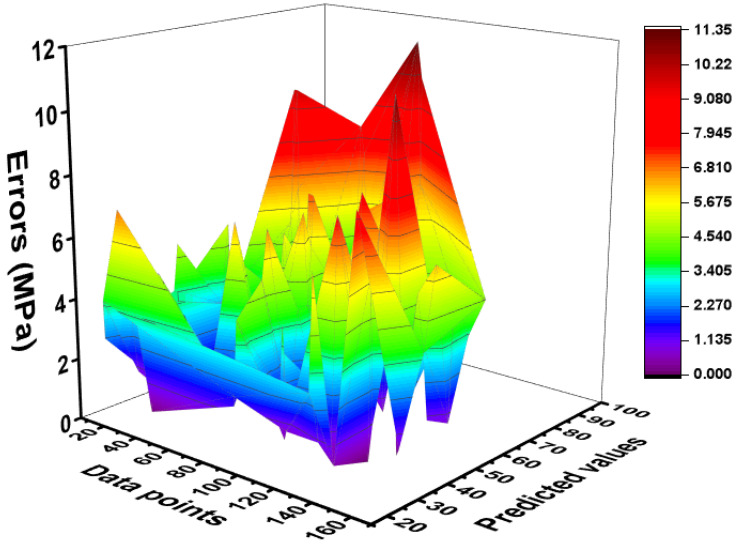
Error graphs of training set of MEP model.

**Figure 10:**
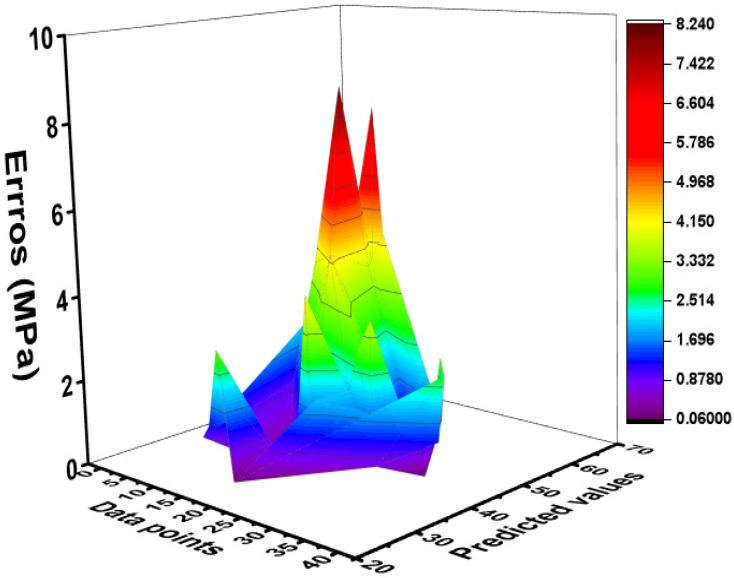
Error graphs of validation set of MEP model.

The created MEP model’s adaptability was further measured by calculating the error distribution between the experimental and predicted values in both datasets (training and validation sets). The error pattern for the training and validation sets is depicted in Figure 9 and Figure 10 for both sets. The deeper red color indicates the greater error levels. The model’s error value is small, indicating that it successfully simulates the compressive strength of CRHA. The whole database is displayed with the absolute error in each data point to see the model’s maximum error percentage, as shown in Figure 10. As can be observed, the model and predicted outputs are quite near, with an average error of 2 MPa and a peak error of less than 6 MPa for the compressive strength of CRHA. Additionally, the frequency of occurrence of maximal error is rather low. It has been discovered that around 80% of CRHA results estimated compressive strengths have an inaccuracy of less than 4 MPa.

### 5.2. Performance Evaluation of MEP Model

According to Iqbal et al. [43], the database-to-input ratio should be at least three for good models and preferably greater than five for perfect models. The ratio is substantially greater in this research, at 32. Table 5 exhibit the statistical parameters for the validation and training sets for the MEP model. These results demonstrate that the models have been trained efficiently and that there is a strong correlation between expected and experimental output with low error levels. The MAE, RMSE, and RE values for the training set of the MEP model are 3.067, 3.843, and 0.047, respectively, while the values for the validation phase are 2.317, 3.406, and 0.048. The statistical measurements are nearly the same for the validation and training sets, demonstrating a greater capability for generalization and the ability to predict trustworthy outcomes for previously unknown data. As seen in Table 5, the ρ of the MEP projected model approaches zero (zero for ideal model). The OBF values of 0.04 adequately solved the issue of data overfitting.

### 5.3. External Validation

External validation of the MEP model was also examined, owing to its substantially improved efficiency, which is shown in Table 6. As per literature, at least one regression slope line (k or k′) going through the origin must approach one [62]. The performance indices must have values less than 0.1. For the situation of optimal moisture content, the requirement of additional external validation, namely, R_m_ > 0.5, is met [63,64,65]. Additionally, the squared correlation coefficient (${R^{\prime }}_{o}^{2}$) between the estimated and experimental datasets, as well as the correlation coefficient ($R_{o}^{2}$) between the experimental and estimated values, must approach one [66,67,68]. As seen in Table 6, the suggested MEP model meets nearly all the stated requirements, which is consistent with the findings of existing literature and recommendations [69,70,71,72].

### 5.4. 10-K Fold Cross Validation

**Figure 11:**
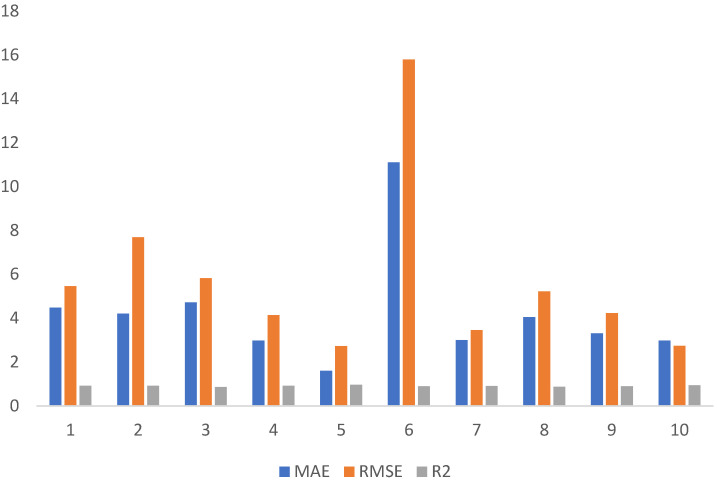
Results of K-fold validation.

A desired level of accuracy is required for the validity of prediction models. The 10 K-fold cross-validation method is used to ensure the accuracy of the model by shuffling the available data. By using this technique, the bias associated with a random sampling of training data set is minimized. This technique divides the experimental data samples into ten equal subsets and utilizes the nine subsets for developing and shaping the strong learner. Meanwhile, the last subset is utilized to gauge the validity of the developed model. The validation process repeats for ten times, and at the end, the average accuracy is obtained from the ten times repetition. The generalization performance and the reliability of the model are well represented by 10 K-fold cross-validations [65]. The cross-validation tests for individual MEP model are represented in Figure 11. The results of 10 K-fold cross-validations are assessed by using the coefficient of determinant, R^2^ (regression tool) along with MAE and RMSE (statistical error tools) as shown in Table 7. In Figure 11, fluctuation in the value R^2^ is observed for the 10 K-fold validation of different ML techniques, but still, a high level of accuracy is maintained in each fold. The accuracy of the cross-validation was also assessed in terms of MAE and RMSE and is given in Figure 11, respectively. The average value of MAE for is 4.2 MPa, respectively, as shown in Figure 11. 

Figure 11 shows the RMSE values of 10 K-fold validation and gives an average value of 5.7 MPa, respectively. The results of the 10 K-fold cross-validation method reflect the accuracy and reliability of the concerned developed models.

### 5.5. Explanation Using MEP Model

**Figure 12:**
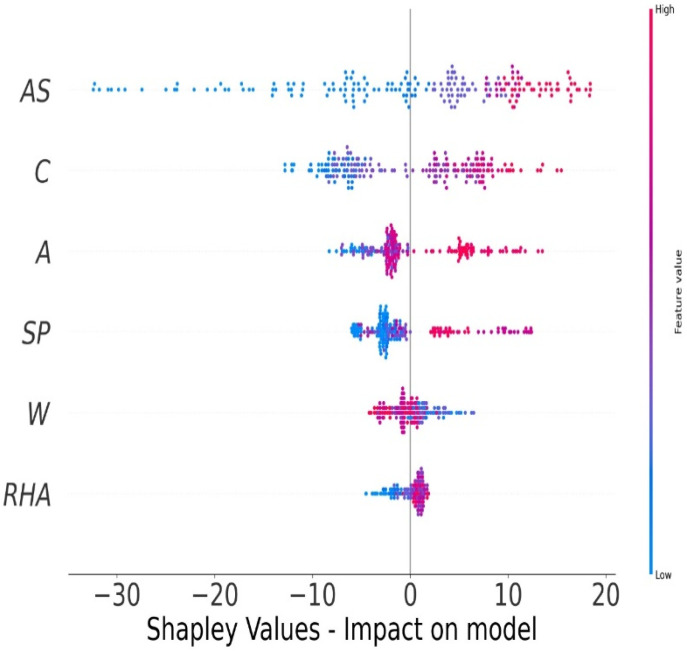
Shapley values of MEP model.

A detailed explanation of the machine learning model, as well as the feature correlations and interactions, is performed. To begin, better global depictions of feature impacts are created by aggregating local descriptions from the SHAP tree integrator over the whole dataset. Figure 12 illustrates a SHAP summary graphic in which each mark corresponds to a single data point in the dataset. The dots along the *x*-axis represent the effect of each feature values on the compressive strength of CRHA prediction. The marks are heaped together to demonstrate the density of several dots landing at the same *x*-axis point. According to Figure 12, the top three characteristics that have the most effect on compressive strength of CRHA prediction, in order of importance, are the age of concrete (AS), the amount of cement (C), and the amount of aggregate (A).

**Figure 13:**
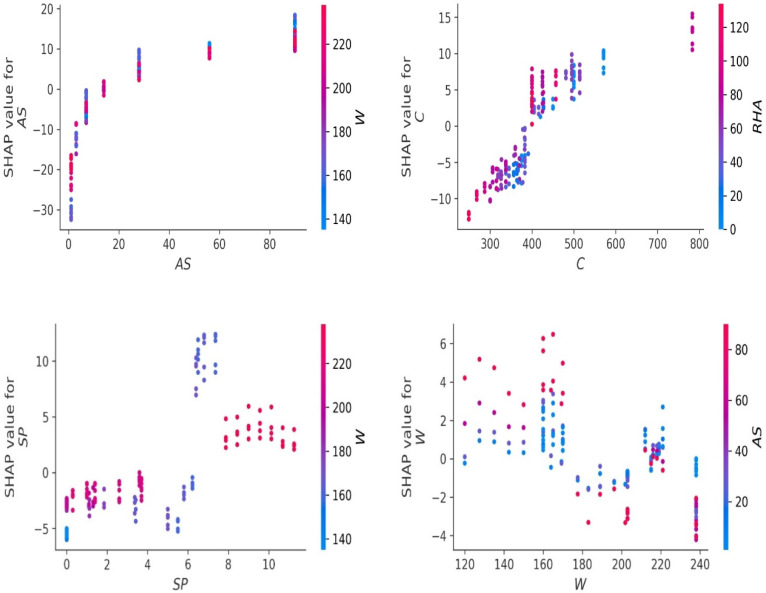
Feature reliance of the model.

Figure 13 illustrates the feature reliance on the machine learning model in further depth by evaluating every single value in the dataset independently. On the *x*- and *y*-axes, the feature values and their related SHAP values are shown. The plots are additionally enhanced by feature interactions (shown by color bars) that indicate the combined influence of many features. One must keep in mind that SHAP values do not indicate causal linkages but rather characterize the model’s behavior. A greater SHAP value implies that the model is attempting to forecast higher compressive strengths from the associated feature values. Similarly, a SHAP value less than zero indicates that the model is seeking to reduce the predicted compressive strength. These microscopic representations demonstrate interactions between various feature pairs impact the related SHAP values, which correlate to the comparable compressive strength values.

Historically, AIA were mostly viewed as black boxes that served as a significant barrier between research and practice [73,74,75]. Because of AIA’s lack of explainability and credibility, practitioners avoid it [75]. However, due to the improved predictability and explainability of the MEP model described in this study, it may be used by a broader range of experts to make some real-world judgments. This amount of data regarding the composition versus strength connection of concrete enhances one’s comprehension of the concrete’s nature and the optimization of the concrete mixture.

### 5.6. Sensitivity Analysis

**Figure 14:**
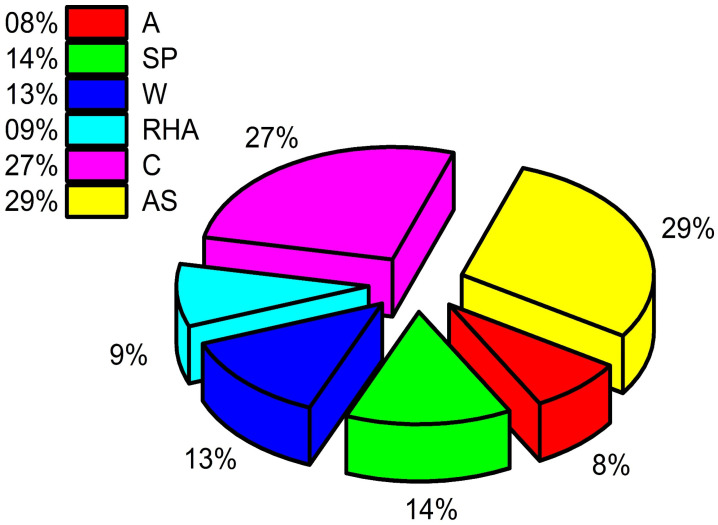
Sensitivity analysis of CRHA concrete.

Figure 14 demonstrates that each parameter is crucial for predicting the compressive strength of CRHA. According to sensitivity analysis, cement and age have a significant part in the total contribution to compressive strength, which is greater than fifty percent. Age of concrete (AS) provides around 29.47 percent, whereas cement quantity (C) contributes approximately 27.93 percent. The remaining four factors, namely RHA, water (W), SP, and aggregate (A), contribute about 8.26%, 12.85%, 13.49%, and 7.99%, respectively.

## 6. Conclusions

Experts have been examining several AIA techniques for predicting the compressive strength of CRHA as feasible alternatives to the highly time-consuming and costly experimental compression testing. However, little effort has been made to improve the predictive powers and explainability of these commercial AIAs, which function as a significant barrier between research and practice, since practitioners avoid adopting AIA owing to their lack of understandability and reliability. To address this, an MEP model is employed to increase the predictability of the compressive strength of CRHA’s. Advanced AIA principles such as model pipelining, model optimization, and feature selection via cross-validation are employed to help in the generation of more accurate models to forecast the compressive strength of CRHA. A comparison of the findings demonstrates that the created model generates the most precise prediction when compared to previously published models over the last two decades.

It is proved that the created MEP model generates verifying data (not available in the current literature) regarding the feature impacts, dependencies, and interactions with the compressive strength of CRHA. The core concept of this study was to explain a prediction model by calculating the contribution of each feature to the prediction of CRHA’s compressive strength. In addition, the relationship between different variables affecting the strength of CRHA is calculated using SHAP analysis. It was discovered that the most significant factors impacting the compressive strength of CRHA are the age of concrete (AS), amount of cement (C), and the amount of aggregate (A). Furthermore, the dependency factors and relationship between different variables may help in future research to make a novel CRHA mix design as per the requirement of the site without compromising on cost, mechanical properties, available time, and availability of the mix ingredients.

### Future Recommendation

The CRHA can effectively replace OPC concrete. Recommendation: comprehensive research of CRHA that includes more parameters. Including more input parameters and expanding the database can yield more trustworthy results for more generic expressions. These parameters should include resistance to acid attack and high temperature, sulphate and chloride resistance, and corrosion. For additional predictions, sophisticated techniques such as particle swarm programming and ensemble methods can be utilized.

ML approaches can be used with heuristic methods, such as the whale optimization, ant colony optimization, and PSO, for improved outcomes. These procedures may then be compared to those utilized in this investigation. In addition, MEP is an expanded and enhanced version of GEP. It is necessary to apply and analyze Honeybee algorithm to overcome the limits of ensemble algorithms.

## Figures and Tables

**Table 1:** Coefficient of correlation (R) for explanatory variables.

	AS * (Day)	C * (kg/m^3^)	RHA * (kg/m^3^)	W * (kg/m^3^)	SP * (kg/m^3^)	A * (kg/m^3^)	CS (MPa)
AS (day)	1.00						
C30 (kg/m^3^)	−0.11	1.00					
RHA (kg/m^3^)	−0.03	−0.22	1.00				
W (kg/m^3^)	0.01	0.08	0.14	1.00			
SP (kg/m^3^)	0.00	0.25	−0.02	0.27	1.00		
A (kg/m^3^)	−0.06	−0.24	−0.14	−0.55	−0.21	1.00	
CS (MPa)	0.49	0.37	−0.02	−0.24	0.30	0.15	1.00

* AS = age of concrete sample, C30 = cement with 30% replacement, W = water, SP = superplasticizer, A = aggregate.

**Table 2:** Statistical description of variables.

Description of Variables	AS (Day)	C (kg/m^3^)	RHA (kg/m^3^)	W (kg/m^3^)	SP (kg/m^3^)	A (kg/m^3^)	CS (MPa)
Mean	34.57	409.02	62.33	193.54	3.34	1621.51	48.14
Median	28.00	400.00	57.00	203.00	1.85	1725.00	45.95
Mode	28.00	400.00	0.00	203.00	0.00	1725.00	47.00
Standard Deviation	33.52	105.47	41.55	31.93	3.52	267.77	17.54
Sample Variance	1123.61	11,124.88	1726.77	1019.71	12.37	71,702.44	307.70
Skewness	0.75	1.55	0.44	−0.42	0.69	−0.74	0.83
Range	89.00	534.00	171.00	118.00	11.25	930.00	88.10
Minimum	1.00	249.00	0.00	120.00	0.00	1040.00	16.00
Maximum	90.00	783.00	171.00	238.00	11.25	1970.00	104.10
Sum	6638.00	78,531.00	11,967.10	37,158.91	640.35	311,330.00	9243.10
Count	192.00	192.00	192.00	192.00	192.00	192.00	192.00

**Table 3:** MEP parameter used in making a model.

Parameters	MEP
Num of subpopulation	20
Subpopulation size	1000
Code length	50
Crossover probability	0.9
Crossover type	Uniform
Mutation probability	0.001
Tournament size	2
Operators	0.5
Variables	0.5
Number of generations	1000
Function set	+, −, ×, /
Terminal set	Problem input
Replication number	10
Error measure	Mean squared error
Problem type	Regression
Simplified	Yes
Random seed	0
Number of runs	10
Number of threads	1

**Table 4:** MEP optimal combination.

Trial No.	No. of Subpopulation	Subpopulation Size	Code Length	No. of Generation	Functions Used	R^2^	RMSE	MAE	RRSE	Time (Min)
MP1	10	200	20	200	+, −, ×, /	0.9275	71.1	48.03	0.2693	0–2
MP2	20		20		+, −, ×, /	0.9448	62.17	41.82	0.2355	
MP3	50		25		+, −, ×, /	0.9454	61.94	45.67	0.2346	
MP4	70		25		+, −, ×, /	0.9233	74.09	47.03	0.2806	
MP5	100		35		+, −, ×, /	0.9221	74.33	46.89	0.2815	
MP6	20	400	35		+, −, ×, /	0.9156	88.17	60.35	0.334	
MP7		600	35		+, −, ×, /	0.9496	59.68	41.9	0.226	
MP10			40	400	+, −, ×, /	0.9614	53.41	38.12	0.2023	15
MP11			40	600	+, −, ×, /	0.9376	66.01	42.78	0.25	25
MP12		1000	50		+, −, ×, /	0.9298	70.13	43.56	0.2656	
MP13			50	1000	+, −, ×, /	0.9362	66.97	45.06	0.2536	45

**Table 5:** Statistical indictors for training and validation set.

Indicators	Training	Validation
R^2^	0.976419	0.971378
R	0.988139	0.985585
RMSE	3.843116	3.406354
MAE	3.067433	2.317413
RRMSE	0.079188	0.072075
RE	0.047253	0.048581
ρ	0.03983	0.0363
OBF	0.04	

**Table 6:** External validation of data.

S. No.	Equation	Condition	MP	Suggested by
1	$R=\frac{\sum_{i=1}^{n}\left(x_{i}-{\bar{x}}_{i}\right)\left(y_{i}-{\bar{y}}_{i}\right)}{\sqrt{\sum_{i=1}^{n}{\left(x_{i}-{\bar{x}}_{i}\right)}^{2}\sum_{i=1}^{n}{\left(y_{i}-{\bar{y}}_{i}\right)}^{2}}}$	R > 0.8	0.98	[63,64,65]
2	$k=\frac{\sum_{i=1}^{n}\left(x_{i}\times y_{i}\right)}{x_{i}^{2}}$	0.85 < k < 1.15	0.975	[62]
3	$k^{\prime }=\frac{\sum_{i=1}^{n}\left(x_{i}\times y_{i}\right)}{y_{i}^{2}}$	0.85 < k′ < 1.15	0.976	
4	$R_{m}=R^{2}\times (1-\sqrt{\left|R^{2}-R_{0}^{2}\right|}$ where $R_{o}^{2}=1-\frac{\sum_{i=1}^{n}{\left(y_{i}-x_{i}^{o}\right)}^{2}}{\sum_{i=1}^{n}{\left(y_{i}-\bar{y_{i}^{o}}\right)}^{2}},\;x_{i}^{o}=k\times y_{i}$ $\;{R^{\prime }}_{o}^{2}=1-\frac{\sum_{i=1}^{n}{\left(x_{i}-y_{i}^{o}\right)}^{2}}{\sum_{i=1}^{n}{\left(x_{i}-\bar{x_{i}^{o}}\right)}^{2}},\;y_{i}^{o}=k^{\prime }\times x_{i}$	R_m_ > 0.5	0.856	[66,67,68]
		$R_{o}^{2}\;≅1$	0.989	[69,70,71,72]
		${R^{\prime }}_{o}^{2}\;≅1$	1.000	

**Table 7:** Statistics for K-fold Validation.

MAE	RMSE	R^2^
4.47	5.4	0.919
4.209	7.68	0.91
4.71	5.82	0.86
2.97	4.14	0.91
1.60	2.71	0.95
11.1	15.	0.89
2.99	3.45	0.90
4.04	5.21	0.87
3.30	4.22	0.89
2.97	2.73	0.93

## Data Availability

The data used in this research has been properly cited and reported in the main text.

## Supplementary Materials

The following supporting information can be downloaded at: https://www.mdpi.com/article/10.3390/ma15113808/s1, Table S1: A database of 192 data points based on the literature review and the published data to build computational equation for predicting the compressive strength of CRHA [52,53,54,55,56,57,58].

## Abbreviations

RHA	Rice husk ash
MEP	Multi-expression programming
CRHA	Concrete made with rice husk ash
SHAP	SHapley Additive exPlanations
OPC	Ordinary Portland cement
AIA	Artificial intelligence algorithms
BBA	Black-box algorithms
EA	Evolutionary algorithms
GA	Genetic algorithm
NN	Neural network
GEP	Gene expression programming
SP	Superplasticizer
C	Amount of cement
W	Amount of water
A	Amount of aggregate
AC	Age of concrete
CS	Compressive strength
RMSE	Root mean square error
R	Coefficient of correlation
MAE	Mean absolute error
R^2^	Coefficient of regression
RRMSE	Relative root mean square error
RE	Relative squared error
ρ	Performance index
OBF	Objective function
NSE	Normalized mean square error

## Appendix A

pg[0] = x[0];
pg[1] = sqrt(pg[0]);
pg[2] = x[1];
pg[3] = x[3];
pg[4] = exp(pg[1]);
pg[5] = pg[1] × pg[2];
pg[6] = pg[4] / pg[3];
pg[7] = pg[0] + pg[3];
pg[8] = pg[2] − pg[7];
pg[9] = x[4];
pg[10] = pg[8] × pg[9];
pg[11] = pg[10] + pg[5];
pg[12] = pg[1] × pg[0];
pg[13] = pg[8] + pg[11];
pg[14] = pow(pg[1], pg[9]);
pg[15] = pg[13] − pg[12];
pg[16] = sqrt(pg[15]);
pg[17] = x[2];
pg[18] = pg[6] + pg[8];
pg[19] = pg[9] × pg[17];
pg[20] = pg[12] / pg[18];
pg[21] = pg[19] + pg[18];
pg[22] = sqrt(pg[11]);
pg[23] = pg[5] / pg[21];
pg[24] = sqrt(pg[14]);
pg[25] = pg[24] × pg[4];
pg[26] = pg[6] − pg[20];
pg[27] = pg[22] / pg[25];
pg[28] = pg[26] / pg[23];
pg[29] = pg[16] − pg[27];
pg[30] = pg[29] + pg[28];
